# Health-related quality of life in adolescents with screening-detected celiac disease, before and one year after diagnosis and initiation of gluten-free diet, a prospective nested case-referent study

**DOI:** 10.1186/1471-2458-13-142

**Published:** 2013-02-16

**Authors:** Katrina Nordyke, Fredrik Norström, Lars Lindholm, Hans Stenlund, Anna Rosén, Anneli Ivarsson

**Affiliations:** 1 Department of Public Health and Clinical Medicine, Epidemiology and Global Health, Umeå University, Umeå, Sweden

**Keywords:** Adolescents, Celiac disease, EQ-5D, Health-related quality of life, Screening, Screening-detected celiac disease

## Abstract

**Background:**

Celiac disease (CD) is a chronic disorder in genetically predisposed individuals in which a small intestinal immune-mediated enteropathy is precipitated by dietary gluten. It can be difficult to diagnose because signs and symptoms may be absent, subtle, or not recognized as CD related and therefore not prompt testing within routine clinical practice. Thus, most people with CD are undiagnosed and a public health intervention, which involves screening the general population, is an option to find those with unrecognized CD. However, how these screening-detected individuals experience the diagnosis and treatment (gluten-free diet) is not fully understood. The aim of this study is to investigate the health-related quality of life (HRQoL) of adolescents with screening-detected CD before and one year after diagnosis and treatment.

**Methods:**

A prospective nested case-referent study was done involving Swedish adolescents who had participated in a CD screening study when they were in the sixth grade and about 12 years old. Screening-detected adolescents (n = 103) and referents without CD who participated in the same screening (n = 483) answered questionnaires at the time of the screening and approximately one year after the screening-detected adolescents had received their diagnosis that included the EQ-5D instrument used to measure health status and report HRQoL.

**Results:**

The HRQoL for the adolescents with screening-detected CD is similar to the referents, both before and one year after diagnosis and initiation of the gluten-free diet, except in the dimension of pain at follow-up. In the pain dimension at follow-up, fewer cases reported problems than referents (12.6% and 21.9% respectively, Adjusted OR 0.50, 95% CI 0.27-0.94). However, a sex stratified analysis revealed that the significant difference was for boys at follow-up, where fewer screening-detected boys reported problems (4.3%) compared to referent boys (18.8%) (Adjusted OR 0.17, 95% CI 0.04-0.73).

**Conclusions:**

The findings of this study suggest that adolescents with unrecognized CD experience similar HRQoL as their peers without CD, both before and one year after diagnosis and initiation of gluten-free diet, except for boys in the dimension of pain at follow-up.

## Background

Celiac disease (CD) is a chronic disorder in genetically predisposed individuals in which a small intestinal immune-mediated enteropathy is precipitated by dietary gluten [1]. The prevalence has generally been suggested to be around 1% [2-4], however more recently it has been shown that the prevalence has risen [3,5,6] and most of the people with CD are actually undiagnosed [7-10]. CD can be difficult to diagnose because the signs and symptoms may be absent, subtle, or not recognized as CD related and therefore not prompt testing within routine clinical practice [11]. The best strategy for finding those with unrecognized CD is still debated, but options include: active case finding through routine clinical practice with a broader consideration for variety in presentation, testing groups at high risk (e.g., first degree relatives of those with CD or those with other autoimmune disorders), or a public health intervention that would involve screening the general population [3,7,10,12,13].

Recently, Aggarwal et al. presented a review of literature that addresses how screening, diagnosing, and treating (gluten-free diet) asymptomatic individuals affects their quality of life [3]. Their overview illustrates that there is a lack of consensus on whether or not the gluten-free diet (GFD) improves the quality of life in “asymptomatic screening-detected” individuals [3]. Research addressing the quality of life (QoL) and health-related quality of life (HRQoL) for people with CD is mostly based on those diagnosed through routine clinical practice or selected for screening because they are considered at high risk [14-28]. The HRQoL for individuals who have received their CD diagnosis from a screening of the general population could differ from these groups and this study focuses on adolescents diagnosed with CD as a result of screening study. The aim of this study is to investigate the HRQoL of adolescents with screening-detected CD before and one year after diagnosis and treatment.

## Method

### Design

A prospective nested case-referent study was done involving adolescents who had participated in a CD screening study. The cases included those with screening-detected CD and referents were chosen from those who tested negative for the CD serological markers. The cases and referents reported their HRQoL at the time of the screening and about one year after the cases received their diagnosis.

### Setting

The CD screening study that the adolescents participated in (ETICS-Exploring the Iceberg of Celiacs in Sweden) took place in 2005–2006 [8]. It involved sixth graders from 5 regions in Sweden when they were about 12 years old. The screening took place in their schools in collaboration with school health systems and regional pediatric departments. Information was provided to parents and children, written consent was obtained from parents, and ethical approval for the study was granted by the Regional Ethical Review board in Umea, Sweden [Dnr UMU 04-156-M].

### Participants and materials

In total, 10 041 children were invited and 7 567 (75%) consented to participate. Blood samples were collected from 7 208 (72%) children who did not already have a CD diagnosis. The blood samples were analyzed for CD serological markers and children with suspected CD were referred to their pediatric department for an intestinal biopsy to confirm the diagnosis [8]. Thereafter, a GFD was recommended and follow-up care was provided according to current clinical standards.

**Figure 1:**
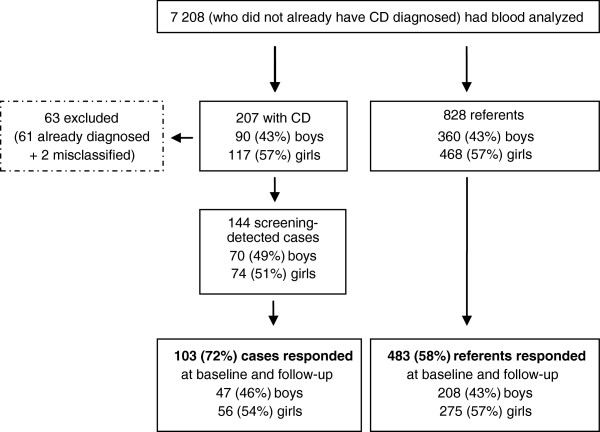
Participants.

At the time the population for this study was selected, there were 145 children with screening-detected/biopsy verified CD and 62 who reported having CD diagnosed prior to the screening and gave a blood sample. After combining these groups with CD (n = 207), 4 referents per child were randomly chosen (n = 828), with the proportion of girls and boys in the referent group to match that of the group with CD. From the 207 children with CD, 2 were found to be without CD, resulting in 61 diagnosed prior to the screening and 144 screening-detected cases (Figure 1).

The participants filled out questionnaires that included the Swedish child-friendly pilot version of the EQ-5D instrument (EQ-5D) [29-31]. The EQ-5D is a generic tool used to measure health status and report HRQoL [32]. It is comprised of two parts, the EQ-5D descriptive system where respondents classify their health status in five dimensions: mobility, self-care, usual activities, pain/discomfort, and anxiety/depression and on level of severity (no problems, moderate problems, or severe problems). The second part of the EQ-5D is a thermometer like visual analogue scale (VAS) where respondents score their health today from worst to best imaginable (0–100) [32,33].

After blood samples were collected, but before the results of the screening were known, the 12-year-olds were given the questionnaires to fill out at school. Baseline EQ-5D responses were received from 138 with screening-detected CD (96% responding) and 797 referents (96% responding). Approximately one year after the screening, follow-up questionnaires were mailed to the homes of those considered cases and referents for this nested-case referent study. Follow-up EQ-5D responses were received from 110 with screening-detected CD (76% responding) and 501 referents (61% responding).

Responses were included for the screening-detected cases and referents when they had provided answers for all five dimensions and on the VAS at both baseline and follow-up (cases n = 103 and referents n = 483) (Figure 1). The median age of the cases at the time of the diagnostic biopsy was 13.2. Because the participants answered the questionnaires approximately one year after the cases had received the diagnosis their median age was 14.6 and we consider them adolescents at follow-up.

### Analysis

Number and proportion of adolescents reporting problems were explored for each dimension. Because few adolescents reported severe problems, levels of severity were collapsed into “no problems” (from level no problems) and “problems” (from levels some problems and severe problems) [33]. The statistical software package SPSS 19 (SPSS Inc., Chicago, IL) was used and statistical significance was defined at the 5% level.

Cross tabulations were done separately for each dimension and for baseline and follow-up to illustrate the proportion reporting problems. Bivariate logistic regression was used to compare the proportion reporting problems for the cases and referents separately at baseline and follow-up, and between the cases and referents at baseline and follow-up, for each dimension. Then, in order to assess and compare crude and adjusted odds ratios (OR) and confidence intervals (CI), multivariate logistic regression analyses were also performed for each dimension. In the logistic regression models, case/referent was the dependent variable and problems at baseline and problems at follow-up were independent variables, separately for the bivariate analyses (Crude OR) and combined in the same model for the multivariate analyses (Adjusted OR). Sex stratified analyses were done for the pain and anxiety dimension, however not for the other dimensions as there were too few reporting problems to motivate further exploration.

The VAS scores of the cases and referents, and the VAS scores for boys and girls within the case and referent groups, were compared using the Mann–Whitney U test. When we compared baseline to follow-up scores in the case and referent groups we used the Wilcoxon signed rank test.

## Results

The HRQoL for the adolescents with screening-detected CD, as reported on the EQ-5D instrument, is similar to the HRQoL of the referents, both before and one year after diagnosis and initiation of the GFD, except for in the dimension of pain at follow-up, where fewer cases reported problems than referents (12.6% and 21.9% respectively, Adjusted OR 0.50, 95% CI 0.27-0.94) (Table 1). However, when this dimension was stratified by sex**,** it was revealed that the difference was between boy cases and referents at follow-up. In the sex stratified results there was a significant difference between the boys in the pain dimension at follow-up, where fewer of the screening-detected boys reported problems (4.3%) than the boy referents (18.8%) (Adjusted OR 0.17, 95% CI 0.04-0.73) (Table 2).

**Table 1:** Adolescents reporting problems (EQ-5D), before and 1 year after screening-detected celiac disease diagnosis, compared to referents without celiac disease

**Dimensions at Baseline and Follow–up**	**Screening-detected CD (cases*n = 103*)**	**Non CD (referents*n = 483*)**	**Odds Ratios (95% CI)**^**a**^	
	**n (%) with problems**	**n (%) with problems**	**Bivariate LR**^**b**^	**Multivariate LR**^**c**^
**Mobility**				
Baseline	3 (2.9)	11 (2.3)	0.78 (0.21-2.84)	1.28 (0.35-4.68)
Follow-up	1 (1.0)	7 (1.4)	0.67 (0.08-5.48)	0.67 (0.08-5.52)
**Self care**				
Baseline	0 0	2 (0.4)	0.00 (0.00-)	0.00 (0.00-)
Follow-up	0 0	2 (0.4)	0.00 (0.00-)	0.00 (0.00-)
**Activity**				
Baseline	3 (2.9)	13 (2.7)	0.92 (0.26-3.30)	1.09 (0.31-3.91)
Follow-up	2 (1.9)	11 (2.3)	0.85 (0.19-3.89)	0.85 (0.18-3.88)
**Pain**				
Baseline	21 (20.4)	95 (19.7)	0.96 (0.56-1.62)	1.18 (0.69-2.02)
Follow-up	**13 (12.6)**	**106 (21.9)**	**0.51 (0.28-0.96)**	**0.50 (0.27-0.94)**
**Anxiety/Depression**				
Baseline	13 (12.6)	52 (10.8)	0.84 (0.44-1.60)	1.37 (0.70-2.69)
Follow-up	15 (14.6)	95 (19.7)	0.70 (0.39-1.26)	0.65 (0.35-1.20)

^a^ Odds ratio (95% confidence interval).

^b^ Bivariate logistic regression using case/referent and problems reported.

^c^ Multivariate logistic regression using case/referent and problems reported at baseline and problems reported at follow-up.

**Table 2:** Total adolescents, boys, and girls reporting problems in pain dimension (EQ-5D), before and 1 year after screening-detected celiac disease diagnosis, compared to referents without celiac disease

**Groups at Baseline and Follow-up**	**Screening-detected CD (cases)**	**Non CD (referents)**	**Odds Ratios (95% CI)**^**a**^	
	**n (%) with problems**	**n (%) with problems**	**Bivariate LR**^**b**^	**Multivariate LR**^**c**^
**Total**	*n = 103*	*n = 483*		
Baseline	21 (20.4)	95 (19.7)	0.96 (0.56-1.62)	1.18 (0.69-2.02)
Follow-up	**13 (12.6)**	**106 (21.9)**	**0.51 (0.28-0.96)**	**0.50 (0.27-0.94)**
**Boys**	*n = 47*	*n = 208*		
Baseline	13 (27.7)	41 (19.7)	1.56 (0.76-3.22)	1.90 (0.90-4.03)
Follow-up	**2 (4.3)**	**39 (18.8)**	**0.19 (0.05-0.83)**	**0.17 (0.04-0.73)**
**Girls**	*n = 56*	*n = 275*		
Baseline	8 (14.3)	54 (19.6)	0.68 (0.31-1.53)	0.72 (0.32-1.62)
Follow-up	11 (19.6)	67 (24.4)	0.76 (0.37-1.55)	0.81 (0.39-1.67)

^a^ Odds ratio (95% confidence interval).

^b^ Bivariate logistic regression using case/referent and problems reported.

^c^ Multivariate logistic regression using case/referent and problems reported at baseline and problems reported at follow-up.

In the anxiety dimension, both the cases and referents had an increase (from baseline to follow-up) in the proportion of adolescents who reported problems, although these were not significant changes (Table 1). When stratified by sex, there was no significant difference in proportion of problems reported for anxiety for the boys or girls between the cases and referents (Table 3).

**Table 3:** Total adolescents, boys, and girls reporting problems in anxiety dimension (EQ-5D), before and 1 year after screening-detected celiac disease diagnosis, compared to referents without celiac disease

**Groups at Baseline and Follow-up**	**Screening-detected CD (cases)**	**Non CD (referents)**	**Odds Ratios (95% CI)**^**a**^	
	**n (%) with problems**	**n (%) with problems**	**Bivariate LR**^**b**^	**Multivariate LR**^**c**^
**Total**	*n = 103*	*n = 483*		
Baseline	13 (12.6)	52 (10.8)	0.84 (0.44-1.60)	1.37 (0.70-2.69)
Follow-up	15 (14.6)	95 (19.7)	0.70 (0.39-1.26)	0.65 (0.35-1.20)
**Boys**	*n = 47*	*n = 208*		
Baseline	4 (8.5)	16 (7.7)	1.12 (0.36-3.51)	1.46 (0.43-4.95)
Follow-up	2 (4.3)	18 (8.7)	0.47 (0.11-2.10)	0.41 (0.08-1.98)
**Girls**	*n = 56*	*n = 275*		
Baseline	9 (16.1)	36 (13.1)	1.27 (0.57-2.81)	1.38 (0.61-3.13)
Follow-up	13 (23.2)	77 (28.0)	0.78 (0.40-1.53)	0.73 (0.37-1.47)

^a^ Odds ratio (95% confidence interval).

^b^ Bivariate logistic regression using case/referent and problems reported.

^c^ Multivariate logistic regression using case/referent and problems reported at baseline and problems reported at follow-up.

Adolescents with screening-detected CD had a median VAS score of 91 at baseline and 90 at follow-up (Table 4), which was not a significant change (Wilcoxon signed rank test, *p* value = 0.92). The referents median VAS score was 90 at baseline and at follow-up (Table 4). Comparisons of cases to referents at baseline and at follow-up showed no significant differences (Mann–Whitney U test, not shown).

**Table 4:** VAS scores of adolescents, before and 1 year after screening-detected celiac disease diagnosis, compared to referents without celiac disease

**VAS scores at Baseline and Follow-up**	**Screening-detected CD (cases)**		**Non CD (referents)**	
	***n = 103***		***n = 483***	
	**Median**	**Quartiles**	**Median**	**Quartiles**
		**25**^**th**^**, 75**^**th**^		**25**^**th**^**, 75**^**th**^
Baseline	91	85, 97	90	80, 99
Follow-up	90	80, 99	90	80, 98

When comparing boys and girls within the case and referent groups, the only significant difference was between the boys and girls in the referent group at follow-up (Mann–Whitney U test, *p* value = 0.01, not shown), in which both report a median of 90 but for the boys and girls the 25^th^ percentile is 85 and 75 (respectively) and the 75^th^ percentile is 98.75 and 97 (respectively).

## Discussion

The HRQoL for adolescents with screening-detected CD, as self-reported on the EQ-5D, is similar to that of their peers, both before and one year after diagnosis and GFD, except in the dimension of pain at follow-up. A sex stratified analysis revealed that this is due to the difference between boy cases and referents at follow-up, in which 4.3% of the screening-detected boys report problems and 18.8% of the referent boys report problems (Adjusted OR 0.17, 95% CI 0.04-0.73) (Table 2).

We present a unique study in which adolescents with screening-detected CD report their HRQoL before and one year after diagnosis and treatment. A strength of our study is that the adolescents report baseline HRQoL before knowledge of their CD diagnosis, unlike many studies in which patients are asked to recall how they felt at the time of their diagnosis. We have previously published baseline data, including participants from the same screening study, in which there was also no significant difference in HRQoL for those with unrecognized CD compared to their peers without CD at the time of the screening [34]. However, in this current study we explore the adolescents’ HRQoL before and after diagnosis and treatment.

Another strength of this study is the fact that the adolescents had their CD detected as a result of a screening study, and not clinically or because they were considered high risk. This means that our results may be more reflective of those living with unrecognized CD, which has been seen as a limitation in other studies that attempt to address the QoL for those with undiagnosed CD [3]. In a review of literature that addresses how screening, diagnosing, and treating “asymptomatic screening-detected individuals” affects their QoL, done by Aggarwal et al. [3], most of the studies involved CD patients who were identified from high risk groups [19,26] or were compared based on what type of symptoms led to their diagnosis; i.e., typical, not typical, or those who reported they had not experienced any symptoms [23,24,28]. However, two of the studies included individuals that could be considered as screened from the general population [3,35,36]. In those studies, the individuals who had “typical” symptoms showed an improvement in QoL scores after one year on the GFD, while those who were supposedly symptom free had scores comparable to healthy controls at baseline and at follow-up, [35,36] similar to the screening-detected adolescents in our study.

Even though the EQ-5D instrument is a validated tool and we have used the Swedish child-friendly pilot version, this tool may have limitations in the context of this study. Perhaps, it is not the ideal tool for capturing problems with subtle symptoms or feelings caused by unrecognized CD. Also, these adolescents may have adapted to their current health situation as normal and at baseline rated their health status as high as possible and similar to their peers. If they did experience improvement after diagnosis and treatment, they would be unable to demonstrate improvement from the high health status previously reported. These screening-detected adolescents were also invited to participate in another follow-up study, where they participated in focus group discussions (n = 31) and wrote narratives (n = 91), and when they were asked specifically about change in well-being after diagnosis, 53.8% reported feeling better [37]. In that study, it was also shown that some of the screening-detected children only realized they had been experiencing symptoms after they had been diagnosed and treated [37].

Although it is beyond the scope of this study to provide an explanation, the finding that fewer screening-detected boys (4.3%) reported problems at follow-up in the dimension of pain than the boy referents (18.8%) is interesting to consider and warrants further investigation. In a previous Swedish study, a doubled risk for symptomatic CD in girls compared to boys was found [38]. In this screening study, the male to female ratio for those with clinically diagnosed CD was 1:2 compared to 1:1 for those with screening-detected CD [8]. This difference reveals that, at the time of the screening, a larger proportion of girls compared to boys had already been diagnosed with CD in routine clinical practice [8]. It could be that the boys with unrecognized CD were further progressed in their disease at baseline because they were not as likely as the girls to have been found in clinical practice. One could speculate that, even though they may not have realized the extent of their problems at baseline, they experienced benefit from the treatment resulting in fewer of these boys reporting problems with pain than the boys in the referent group, a phenomenon which was not captured for the girls. In studies involving adults, men and women have been shown to experience the burden of CD differently [27,39,40]. There is also research that shows men and women access health care differently [41-43] and perhaps the boys were less likely to seek or receive care. It is also a possibility that clinicians expect girls to have a higher risk of developing CD and more readily recognize and diagnose girls.

In our study there were no significant differences in the anxiety dimension between cases and referents, suggesting that for these screening-detected adolescents the CD diagnosis and GFD have not caused excess anxiety (at least at one year follow-up). Other studies have shown that those with a strict GFD can have the same HRQoL as healthy children/adolescents [44,45]. However, in the qualitative study mentioned previously [37], it was revealed that some of these adolescents perceived the GFD and lifestyle changes as inconvenient and causing feelings of stigma while others adapted well to the disease.

## Conclusions

The findings of this study suggest that the adolescents with unrecognized CD experience similar HRQoL as their peers without CD, both before and one year after the diagnosis and initiation of the GFD, except for boys in the dimension of pain at follow-up. The screening-detected boys seem to benefit, because fewer report problems with pain than the boy referents at follow-up, one year after diagnosis and initiation of the GFD. However, further research is needed to explore why the screening-detected boys seem to have a different experience from the referent boys and from the screening-detected girls. If considering general population screening for CD in the future, more research is also needed to learn more about the benefits or drawbacks of early diagnosis, long term consequences of untreated CD, and the health economic implications of population screening.

## Abbreviations

CD: Celiac disease; QoL: Quality of life; HRQoL: Health-related quality of life; GFD: Gluten-free diet; LR: Logistic regression; OR: Odds ratio; CI: Confidence interval.

## Competing interests

The authors declare that they have no competing interests.

## Authors’ contributions

All authors contributed to the conception or design of the study. KN, AI, LL, FN, and HS contributed to the analysis. KN wrote the manuscript and all authors read and gave feedback during drafting and revising the manuscript and approved the final version.

## Pre-publication history

The pre-publication history for this paper can be accessed here:

http://www.biomedcentral.com/1471-2458/13/142/prepub
